# Effects of Pharmacists' Interventions on Appropriateness of Prescribing and Evaluation of the Instruments' (MAI, STOPP and STARTs') Ability to Predict Hospitalization–Analyses from a Randomized Controlled Trial

**DOI:** 10.1371/journal.pone.0062401

**Published:** 2013-05-17

**Authors:** Ulrika Gillespie, Anna Alassaad, Margareta Hammarlund-Udenaes, Claes Mörlin, Dan Henrohn, Maria Bertilsson, Håkan Melhus

**Affiliations:** 1 Division of Pharmacokinetics and Drug Therapy,Department of Pharmaceutical Biosciences, Uppsala University and Uppsala University Hospital, Uppsala, Sweden; 2 Department of Medical Sciences, Uppsala University and Uppsala University Hospital, Uppsala, Sweden; 3 Department of Medicine, Uppsala University Hospital, Uppsala, Sweden; 4 Medical Products Agency, Uppsala, Sweden; 5 Uppsala Clinical Research Center, Uppsala, Sweden; Virginia Commonwealth University, United States of America

## Abstract

**Background:**

Appropriateness of prescribing can be assessed by various measures and screening instruments. The aims of this study were to investigate the effects of pharmacists' interventions on appropriateness of prescribing in elderly patients, and to explore the relationship between these results and hospital care utilization during a 12-month follow-up period.

**Methods:**

The study population from a previous randomized controlled study, in which the effects of a comprehensive pharmacist intervention on re-hospitalization was investigated, was used. The criteria from the instruments MAI, STOPP and START were applied retrospectively to the 368 study patients (intervention group (I) n = 182, control group (C) n = 186). The assessments were done on admission and at discharge to detect differences over time and between the groups. Hospital care consumption was recorded and the association between scores for appropriateness, and hospitalization was analysed.

**Results:**

The number of Potentially Inappropriate Medicines (PIMs) per patient as identified by STOPP was reduced for I but not for C (1.42 to 0.93 vs. 1.46 to 1.66 respectively, p<0.01). The number of Potential Prescription Omissions (PPOs) per patient as identified by START was reduced for I but not for C (0.36 to 0.09 vs. 0.42 to 0.45 respectively, p<0.001). The summated score for MAI was reduced for I but not for C (8.5 to 5.0 and 8.7 to 10.0 respectively, p<0.001). There was a positive association between scores for MAI and STOPP and drug-related readmissions (RR 8–9% and 30–34% respectively). No association was detected between the scores of the tools and total re-visits to hospital.

**Conclusion:**

The interventions significantly improved the appropriateness of prescribing for patients in the intervention group as evaluated by the instruments MAI, STOPP and START. High scores in MAI and STOPP were associated with a higher number of drug-related readmissions.

## Background

Inappropriate prescribing causes substantial morbidity and is a clinical and economical burden for patients and society [1], [2]. It has become an important public-health issue, particularly among the elderly [1], [3], [4]. The question of how to ensure safe effective prescribing for this increasing patient population represents a major challenge for our societies. In this context, there is a growing interest in developing methods of measuring the appropriateness of prescribing. The pharmacist's role in supporting and promoting the rational use of medications has been explored and well described [5] and pharmacist interventions have been shown to positively affect clinical health outcomes such as morbidity and adverse drug events (ADEs) [6]–[9]. A systematic approach can be used to measure appropriate prescribing in elderly patients and several instruments, process measures, have been developed for this purpose. Criterion-based instruments have a drug/disease focus and can be used as checklists, while judgment-based instruments focus on the individual patient [10]. For an instrument to be valid, there should be causal links with important outcomes such as hospital care utilization, presence of ADEs or mortality [11]. The best known and most often used criterion-based instrument, the Beers' criteria [12]–[15], has been well studied regarding its ability to predict mortality, use of health-care services, ADEs and quality of life. Some studies show a positive relationship between inappropriate prescribing and increased mortality, use of health-care services and adverse drug events [16]–[18], whereas others report mixed or negative results [19], [20]. The Screening Tool of Older Persons' Prescriptions (STOPP) and the Screening Tool to Alert doctors to Right Treatment (START) criteria are two relatively new criterion-based instruments [21]–[23]. STOPP measures potentially inappropriate medicines (PIMs) and START potential prescribing omissions (PPOs). Hamilton et al found that the likelihood of a serious avoidable ADE increased significantly when STOPP criteria PIMs were prescribed [24] and Dalleur et al that inappropriate prescribing events (both PIMs and PPOs) were frequent and associated with a substantial number of acute hospital admissions [25]. When STOPP and START were used prospectively in a randomized controlled trial there were however no effects on all-cause mortality, falls or readmissions to hospital [26]. The Medication Appropriatness Index (MAI) [27], [28], a judgment-based instrument, has been used extensively in research [26], [29]–[32]. It has also been used in several studies as a reference when assessing the ability of screening instruments to identify inappropriate prescribing [33], [34]. The association between MAI scores and hospitalization has not yet been consistently demonstrated according to a Cochrane review from 2012 [35]. In summary, the evidence that available instruments are associated with adverse patient health outcomes is equivocal and contradictory. Studies that test the predictive ability of instruments that measure inappropriate prescribing for elderly people are required.

Our research group has previously reported the effects of a comprehensive pharmacist intervention, directed towards patients 80 years or older, on hospitalization and the associated costs [6]. The main findings from that prospective, randomized controlled trial (RCT) were that the addition of pharmacists to the health-care team was cost effective and led to significant reductions in hospital visits (combining visits to the emergency department (ED) and readmissions), visits to the ED, and drug-related readmissions during a 12-month follow-up period. The first aim of this study was to further analyze the results of that RCT with respect to the effects of the pharmacist intervention on the appropriateness of prescribing, as measured by the three instruments MAI, STOPP and START. Data from the RCT on health-care utilization were also analyzed to investigate possible links between the instruments and clinical outcomes; i.e. whether patients who are prescribed inappropriate drugs resulting in high scores on MAI, STOPP and START, are hospitalized to a greater extent than patients receiving more appropriate drug treatment. This was therefore our second aim: to evaluate the instruments in terms of ability to predict hospitalization.

## Methods

### Study design

The prospective RCT comparing patients receiving standard (non-pharmacist) care with those receiving enhanced, more comprehensive services where a pharmacist was part of the health-care team has been described in detail previously [6]. Briefly 400 patients aged 80 years or older were included from two acute internal medicine wards at Uppsala University Hospital. Patients from both wards were randomly assigned to the intervention or control groups. The main elements of the enhanced service provided by the clinical pharmacists to the intervention group patients were: a medication reconciliation performed on admission and at discharge, a drug review where identified drug-related problems were orally communicated to and discussed with the physician in charge, follow-up on drug alterations, patient education, communication of treatment plan to primary care representatives, and follow-up phone calls to the patient after discharge. The pharmacist intervention was standardized but the drug review procedure did not include consistent use of any of the existing instruments. Each participant gave written informed consent, and the study protocol was approved by the Uppsala regional ethics committee. Trial Registration: clinicaltrials.gov Identifier: NCT00661310.

### Description of instruments

STOPP consists of 65 commonly encountered instances of potentially inappropriate prescribing in older people, including drug-disease interactions, irrational prescribing and drugs that are known to increase risks (eg. fall, cognitive decline) for the elderly. The criteria identify Potentially Inappropriate Medications (PIMs). START deals with the problem of underprescribing, i.e. failure to prescribe drugs that are indicated, and consists of a list of 22 specific, evidence-based prescribing indicators that each detects Potential Prescription Omissions (PPOs). Both STOPP and START are arranged according to relevant physiological systems for ease of use and each PIM and PPO generates one point; i.e. the scoring is not weighted. In this study, the numbers of PIMs and PPOs were measured on admission and at discharge.

MAI contains 10 questions that are applied to each drug. The questions, formulated without reference to specific drugs or diseases included: *Is there an indication for the drug? Is the dosage correct? Is the drug the least expensive alternative?* When the answer to the question indicated inappropriateness, a score was assigned to the drug. The questions had weighted scores; *lack of effectiveness* scored 3 points, for example, while *drug-drug interactions* scored 2 points and *duplication of drugs* scored 1 point. An MAI score for each drug, the “drug score”, was calculated by weighting and adding the scores. A summated MAI score was also obtained for each patient, the “patient score”, by adding the scores for each drug for that patient's regimen.

For all three instruments, a higher score indicated inappropriate prescribing.

### Study design for the assessment of appropriateness of prescribing

One clinically experienced pharmacist (UG) applied the criteria from MAI, STOPP and START retrospectively to data from the electronic case notes of the 368 included patients (27 of the original 400 patients died during the index admission and 5 patients wished to be excluded from the trial – the drug lists of these patients were not assessed in the study). The patients' medication administration records (MARs) from all hospital visits, laboratory data and electronic medical notes (from primary and hospital care) were available. All drugs were entered into an interaction database (Swedish Finnish Interaction X-referencing, SFINX, http://www.janusinfo.se/sfinx/interactions/index_menus.jsp) to make sure no clinically relevant drug-drug interactions were missed. The patients' drug therapy was assessed twice: on the first day of the hospital stay (after admission and before any recommendations from the pharmacists had been presented to the physicians) and on the day of discharge from hospital. All prescribed drugs with pharmacologically active ingredients were included in the analyses; thus prescribed preparations such as moisturizing creams and saline for catheter flushing were excluded based on this rule. The renal function of all patients was estimated using the Cockroft and Gault equation.

STOPP and START were used according to detailed instructions published by the developers of the instruments. The MAI comes with comprehensive instructions using examples to illustrate the rationale for judgments and scoring. All instructions were carefully followed during the assessments. Data about hospital care and whether the admissions were classified as drug-related had been obtained in the previous study from the hospital administration records and the medical records, respectively. The data were analyzed four years after the original study had closed, in two steps. The control and intervention groups were compared with each other to assess the pharmacists' intervention, and data from the two groups were analyzed together in order to increase the power of the assessment in evaluating the instruments.

### Outcome measurements

For the first objective of this study, the outcome measures were scores for appropriateness of prescribing (using STOPP, START and MAI) on admission and at discharge. The results for the intervention group were compared to those for the control group. For the second aim of the study, the data were analyzed without taking the group assignment into consideration. The primary outcome measure was the extent of utilization of hospital-based care (the number of visits to the ED and readmissions) during the 12 months after the index admission. The secondary outcome measure was the number of drug-related readmissions to hospital during the same period.

### Statistical Analysis

Comparisons between the intervention and control groups with respect to changes from admission to discharge in the scores of the different instruments were performed, using rank analysis of covariance [36], [37] with score at admission as a covariate. The association between the scores and the number of hospitalizations was explored through negative binomial models using the logarithm of time spent outside the hospital as an offset. Unadjusted models were developed, along with models adjusted for baseline covariates: age (continuous), gender (male/female), weight (continuous), social support (spouse or partner/residential home/none), and medical history: heart failure, diabetes, pulmonary disease, arrythmias, malignant disease, coronary artery disease, cerebral vascular lesion, myocardial infarct, hypertension, dementia (yes/no). All statistical analyses were performed using SAS 9.2. For all tests, p-values <0.05 were considered statistically significant.

## Results

The control and intervention groups were well balanced regarding age, gender, diagnoses and social circumstances (Table 1).

**Table 1:** Baseline characteristics.

	Intervention Group (n = 182)	Control Group (n = 186)	Total (n = 368)
Gender, n (%)			
- Female	105 (57.7)	111 (59.7)	216 (58.7)
- Male	77 (42.3)	75 (40.3)	152 (41.3)
Age, years, mean (SD)	86.4 (4.2)	87.1 (4.1)	86.7 (4.1)
Body weight, kg, mean (SD)			
- Female	61.9 (13.8)	60.7 (13.0)	61.3 (13.3)
- Male	70.6 (12.1)	72.1 (12.9)	71.3 (12.5)
Creatinine clearance, ml/min, mean (SD)	40.6 (19.9)	39.9 (17.1)	40.3 (18.5)
Daily prescription medications, mean (SD)	8.7 (4.5)	7.3 (4.4)	8.1 (4.5)
Social support, n (%)			
- Spouse/partner	54 (29.7)	50 (26.9)	104 (28.3)
- Residential home	33 (18.1)	34 (18.3)	67 (18.2)
- None	95 (52.2)	102 (54.8)	197 (53.5)
Duration of admission (index), days, mean (SD)	11.9 (13.0)	10.5 (9.3)	11.2 (11.3)
Heart failure, n (%)	64 (35.2)	52 (28.0)	116 (31.5)
Diabetes, n (%)	48 (26.4)	39 (21.0)	87 (23.6)
Pulmonary disease, n (%)	23 (12.6)	21 (11.3)	44 (12.0)
Arrythmia, n (%)	63 (34.6)	62 (33.3)	125 (34.0)
Malignant disease (past and present), n (%)	28 (15.4)	26 (14.0)	54 (14.7)
Coronary artery disease, n (%)	61 (33.5)	53 (28.5)	114 (31.0)
Cerebral vascular lesion (past), n (%)	38 (20.9)	19 (10.2)	57 (15.5)
Myocardial infarction, n (%)	45 (24.7)	42 (22.6)	87 (23.6)
Hypertension, n (%)	77 (42.3)	70 (37.6)	147 (39.9)
Dementia, n (%)	20 (11.0)	27 (14.5)	47 (12.8)

The change from admission to discharge in MAI, STOPP and START scores differed significantly between the intervention and control groups. On average, the intervention group had lower scores at discharge than admission, while the control group had higher or unchanged scores at discharge compared to admission (Table 2). The results were not normally distributed.

**Table 2:** Scores on admission and at discharge and change from admission.

Instrument		Intervention group (n = 182)			Control group (n = 186)			p-value
		Admission	Discharge	Change from admission^d^	Admission	Discharge	Change from admission^d^	
MAI^a^	Mean (SD)	8.5 (6.8)	5.0 (4.2)	−3.5 (5.1)	8.7 (7.3)	10.0 (7.3)	1.3 (3.1)	p<0.001
	Median (Min, Max)	8 (0–35)	5 (0–20)	−2 (−26–8)	7 (0–34)	8.5 (0–32)	0 (−7–13)	
STOPP^b^	Mean (SD)	1.4 (1.5)	0.9 (1.0)	−0.5 (1.0)	1.5 (1.5)	1.7 (1.5)	0.2 (0.7)	p<0.001
	Median (Min, Max)	1 (0–7)	1 (0–5)	0 (−4–2)	1 (0–7)	1 (0–8)	0 (−3–3)	
START^c^	Mean (SD)	0.4 (0.7)	0.1 (0.3)	−0.3 (0.6)	0.4 (0.7)	0.5 (0.7)	0 (0.4)	p<0.001
	Median (Min, Max)	0 (0–4)	0 (0–2)	0 (−4–0)	0 (0–3)	0 (0–3)	0 (−1–2)	

SD, Standard deviation.

a Summated MAI score per patient.

b Number of PIMs per patient.

c Number of PPOs per patient.

d Change from admission calculated as Score at discharge-Score on admission.

e p-values from rank analysis of covariance for the effect of group status (Intervention or Control) on change from admission, adjusted for the score on admission.

**Figure 1:**
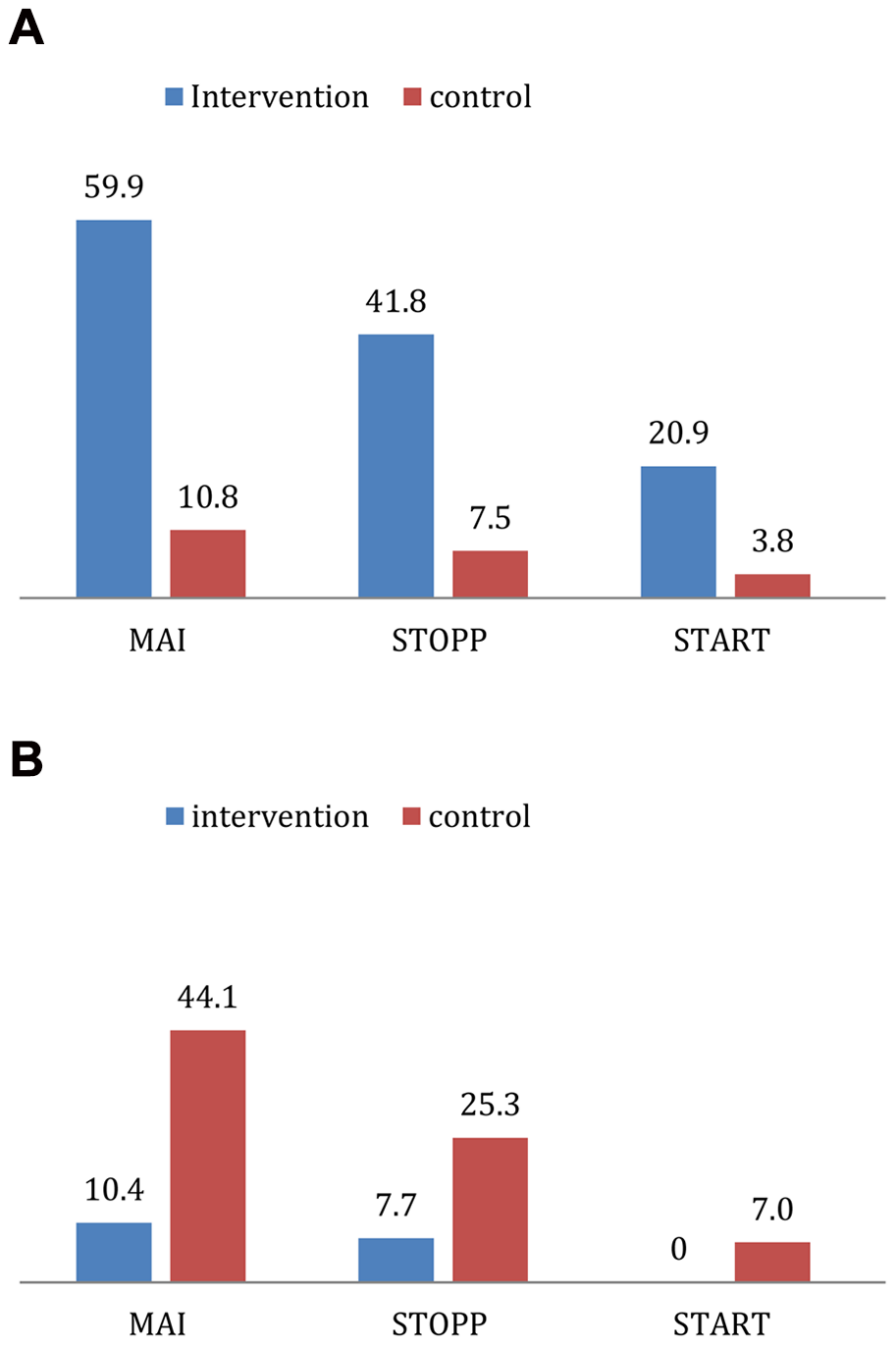
**A.** Percentage of patients with improved scores. **B.** Percentage of patients with deteriorated scores.

Between admission and discharge, the MAI, STOPP and START scores improved in 60%, 42% and 21%, respectively, in the intervention group compared to 11%, 8% and 4% of patients, respectively, in the control group (Figure 1A). In contrast, the MAI, STOPP and START scores deteriorated in 44%, 25% and 7% of patients in the control group, compared with 10%, 8% and 0% in the intervention group (Figure 1B).

For the 368 patients, none of the scores for MAI, STOPP or START were associated with the primary clinical outcomes, involving the total number of visits to hospital or the number of readmissions (Table 3). However, when the unadjusted model was used, the scores for MAI were associated with the number of readmissions with a rate ratio (RR) of 1.02 (Table 3).

**Table 3:** Effect of MAI, START, STOPP on number of total visits to hospital, number of readmissions and number of drug-related readmissions (N = 368).

	Number of total visits to hospital	Number of readmissions	Number of drug-related readmissions
Model^a^	RR (95% CI)^b^	RR (95% CI)^b^	RR (95% CI)^b^
**MAI**			
Unadjusted	1.02 (1.00–1.03), p = 0.053	1.02 (1.00–1.04), p<0.05	1.08 (1.03–1.13), p<0.001
Adjusted	1.02 (1.00–1.03), p = 0.058	1.02 (1.00–1.04), p = 0.060	1.09 (1.04–1.14), p<0.001
**STOPP**			
Unadjusted	1.05 (0.97–1.14), p = 0.24	1.09 (0.99–1.19), p = 0.07	1.30 (1.03–1.63), p<0.05
Adjusted	1.05 (0.97–1.15), p = 0.23	1.06 (0.97–1.16), p = 0.20	1.34 (1.05–1.70), p<0.05
**START**			
Unadjusted	1.05 (0.87–1.28), p = 0.60	1.17 (0.95–1.44), p = 0.14	1.49 (0.92–2.39), p = 0.10
Adjusted	1.09 (0.90–1.32), p = 0.39	1.16 (0.95–1.42), p = 0.14	1.49 (0.91–2.45), p = 0.11

a Negative binomial regressions. Adjusted models include age, gender, weight, social support and medical history.

b RR, Rate ratio; CI, Confidence interval.

In contrast, scores for both MAI and STOPP correlated with drug-related readmissions (Table 3). For MAI (using the adjusted model), an increase in score of one point resulted in a 9% increase in the risk of a drug-related admission. For STOPP (using the adjusted model), an increase of one point was associated with a 34% increase in the risk of a drug-related readmission.

There was no association between the instruments and visits to the ED (data not shown).

## Comment

Improvements in the appropriateness of prescribing, defined by process measures, by introducing a pharmacist intervention have been demonstrated in several previous studies [29]–[31], [38]. This study is important in that, as well as demonstrating the effect of the pharmacist intervention on the quality of prescribing, it explores the link between the three process measures used in the quality assessment and clinical health outcomes. Our analyses confirm that the pharmacist intervention in the RCT substantially improved the appropriateness of prescribing, as measured by the established instruments MAI, STOPP and START. The main finding of this study, was the association between higher scores on the MAI and STOPP instruments (indicating inappropriate prescribing) at discharge and a higher frequency of drug-related hospital readmissions. However, there was no relationship between the scores and revisits to hospital (sum of visits to ED and readmissions).

Of the available criterion-based instruments available, we chose to use STOPP and START since they are recently developed, validated instruments, which have not yet been extensively tested but which appear promising in many ways; they are organized according to therapeutic area and they can be quickly and easily applied [21]. When used together, these instruments cover most pharmacological aspects of inappropriate prescribing, including omission of drugs. In a study where both STOPP and Beer's criteria were applied, it was shown that STOPP identified a significantly higher proportion of patients requiring hospitalization as a result of PIM-related adverse events than the Beers' criteria [39]. We also wanted to use a judgment-based instrument and MAI was chosen.

Instruments for assessing the quality of prescribing should ideally be valid, reliable, sensitive, specific, and feasible [40], [41]. However, there is an inherited tension between these aims and they need to be balanced against each other. Generally, criterion-based instruments are highly reliable and do not depend on the knowledge of the user, but their content is often subject to controversy. Judgment-based instruments, on the other hand, often have high validity since they are based on information about the individual patient but the inter- and intra-rater reliability can be challenged since the researcher needs to be clinically experienced and have access to all relevant patient data. The strengths and weaknesses of each instrument should be taken into consideration both when choosing a method for assessment and when interpreting the results. In our study, none of the scores for the MAI, STOPP or START scores showed an association with revisits to hospital and, therefore, it cannot be suggested that any one of them has a higher predictive ability on the chosen primary outcome measure than the others.

Although STOPP and START were developed to complement each other, the creators of the instruments have, to our knowledge, not used or analyzed the combined scores. In our study, when the PIMs and PPOs were combined, the vast majority (84%) of points for inappropriateness at discharge were from the STOPP criteria. Because the instruments were not equally represented, we chose not to combine them in a single measure, although it was tempting to include all aspects of appropriate prescribing into one score. It is also important to consider that the MAI does not take underprescribing into account and therefore does not cover all aspects of inappropriate prescribing. An area for future research is therefore to find ways to combine these measures in order to use a multidimensional approach.

In the control group, the proportion of patients with deteriorated MAI, STOPP and START scores at discharge was larger than the proportion with improved scores. A possible reason for this could be that the focus was on treating the current cause of admission, and hence drugs were added without a thorough review of previous, on-going prescriptions. The situation was the opposite for the intervention group, with more patients having improved scores at discharge. This confirms that the introduction of a pharmacist to the health-care team encourages prescribing that takes all the diagnoses and other medications into account.

The association between higher scores in STOPP and MAI and an increase in the number of drug-related hospital admissions was statistically significant with a RR of 1.34 for STOPP and 1.04 for MAI. Are these findings clinically relevant? On average, the MAI scores for patients in the intervention group were reduced by 3.5, which indicated a 31.5% lower risk of a drug-related readmission at follow-up. Similarly, the mean reduction of 0.5 in STOPP score in the intervention group indicated a 17% lower risk of a drug-related readmission at follow-up. Previous studies have used drug-related revisits to hospital as an outcome measure for interventions that aim to improve quality of prescribing [29], [42]. Because of the lack of a reliable procedure for identification of drug-related admissions it is, however, difficult to compare these results. In our RCT, the results showed that the pharmacist intervention reduced the number of drug-related readmissions by as much as 80%, which indicates that these admissions could serve as the main target of a pharmacist intervention. In this study, we demonstrated a link between MAI and STOPP scores and the number of drug-related readmissions, which underscores the clinical importance of appropriate prescribing. It also sheds new light on previous studies using the results of these instruments as outcome measures without confirming a link to health outcomes.

The MAI, STOPP and START assessments were conducted by a single pharmacist. This limits the reproducibility of the study, but it also resulted in a more uniform assessment. Our study only included patients aged 80 years or older, while most other similar studies have included patients aged 65 years or older. This impedes comparison of results. Barry et al. found that approximately 57% of elderly patients admitted to hospital had at least one appropriate drug omitted, as assessed by START [23]. In our study, this number was 30%. This difference may be the result of the significantly greater age of the patients in our study (mean age 87 vs. 78 years), in that they were thus more likely to have co-morbidities, with the result that clinically indicated drugs could become contra-indicated.

Since the clinical pharmacist intervention in the RCT consisted of a number of elements and the screening instruments only evaluate the drug review part of the intervention, the reduction in hospital visits that was observed cannot be assumed to be directly due to the MAI, STOPP and START scores. The interventions that aimed to increase the patients' knowledge of and adherence to drug treatment and the reduction in medication errors were for example not taken into consideration. The key elements of the RCT, that were associated with improvements to the appropriateness of prescribing, were the focus on and knowledge of prescribing for the elderly that was brought into the care team by the clinical pharmacists, the close working relationship among doctors, nurses and pharmacists and the extensive communication with the patients. In order to improve the overall quality of drug use, it is necessary not only to focus on pharmacological appropriateness but also to consider the wants and needs of the individual patient. This is most important to keep in mind, both when designing drug review interventions and when assessing the appropriateness of prescribing.

There is currently no validated instrument that covers all relevant aspects of appropriate prescribing. The three instruments used in this study all focus on different aspects of appropriate prescribing but none of them take the patients' views, wishes or adherence to treatment into consideration. However, when used together, the three instruments present a multidimensional assessment that evaluates aspects of both under-, over-, and mis-prescribing.

## Conclusion

The addition of a comprehensive pharmacist service to standard care significantly improved the appropriateness of prescribing for patients in the intervention group that participated in the RCT, as evaluated by the screening instruments MAI, STOPP and START. High MAI and STOPP scores at discharge were associated with a higher number of drug-related readmissions; however, no statistically significant relationship was found between the scores and the total number of re-visits to hospital during a 12-month follow-up period.
